# Recent Advances in the Development and Utilization of Nanoparticles for the Management of Malignant Solid Tumors

**DOI:** 10.7759/cureus.70312

**Published:** 2024-09-27

**Authors:** Dhanashri D Chavan, Rohit R Bhosale, Vandana M Thorat, Amol S Shete, Sarika J Patil, Devkumar D Tiwari

**Affiliations:** 1 Department of Pharmacology, Krishna Institute of Medical Sciences, Krishna Vishwa Vidyapeeth (Deemed to be University), Malkapur, IND; 2 Department of Pharmaceutics, Krishna Foundation's Jaywant Institute of Pharmacy, Wathar, IND; 3 Department of Pharmaceutics, Krishna Institute of Pharmacy, Krishna Vishwa Vidyapeeth (Deemed to be University), Malkapur, IND

**Keywords:** cancer, drug delivery, malignant solid tumors, nanocarriers, nanoparticles, nanotechnology

## Abstract

The purpose of nanotechnology-based drug delivery systems or novel drug delivery systems is to improve the effectiveness of therapy, and their promising properties have led to their increasing significance in the management of cancer. The researchers have primarily focused on designing novel nanocarriers, like nanoparticles (NPs), that can effectively deliver drugs to target cells and respond specifically to conditions particular to cancer. Whether passive or active targeting, these nanocarriers can deliver therapeutic cargoes to the tumor site to release the drug from the drug delivery systems. The purpose of this study is to provide recent scientific literature and key findings to researchers as well as the scientific community from the medical and pharmaceutical domains by reporting current advancements in the development of NPs for the treatment of different malignant solid tumors, such as colorectal, pancreatic, prostate, and cervical cancer.

## Introduction and background

Cancer stands out as a major public health challenge worldwide, ranking as the second leading cause of mortality. Cancer is generally characterized by abnormal cell growth and the potential to invade nearby tissues. Extensive research has sought to pinpoint the various risk factors that can lead to cancer. While some cancers are closely tied to environmental exposures, such as radiation and pollution, lifestyle choices also play a pivotal role. Unhealthy habits like a poor diet, smoking, high stress levels, and a lack of physical activity can greatly increase the risk of developing cancer [1,2]. While it is clear that external factors contribute significantly to cancer, understanding the genetic side of the equation is more complicated. This includes examining mutations in proto-oncogenes, changes in the expression of tumor suppressor genes, and the genes involved in DNA repair. Surprisingly, only a small fraction of about 5%-10% of cancer cases can be traced back to inherited genetic traits. Moreover, age is another crucial factor, and the risk of developing various cancers rises as people age [3]. Cancer treatment typically involves several standard methods, including surgery, chemotherapy, radiation, targeted therapies, immunotherapy, and hormone treatments [4-6]. While chemotherapy and radiation are effective in halting cell growth and killing cancer cells, they often come with significant side effects and a high chance of recurrence [7]. Common adverse effects include neuropathy, bone marrow suppression, gastrointestinal issues, skin reactions, hair loss, and fatigue. Additionally, certain drugs can lead to specific complications, such as cardiotoxicity associated with anthracyclines and pulmonary toxicity linked to bleomycin [8].

The emergence of targeted therapies has significantly advanced the field of precision medicine [9]. However, challenges remain, particularly in the form of unavoidable side effects like multidrug resistance, which can diminish the effectiveness of treatment [8,9]. On the other hand, immunotherapy has shown promising outcomes in treating primary tumors and reducing the likelihood of metastasis and recurrence [10]. Despite these benefits, a notable concern is the potential for autoimmune disorders as a side effect of immunotherapy. Research indicates immunotherapy is less effective for solid tumors than lymphomas [11]. This is partly due to solid tumors' unique extracellular matrix (ECM), which poses a barrier to immune cell infiltration [12]. Furthermore, the latest targeted therapies and immunotherapies disrupt critical signaling pathways essential for both tumor behavior and the normal functioning of the skin's epidermis and dermis, leading to dermatologic adverse events [13].

Considering all these specifics, there has been a growing push in recent years to develop innovative strategies to achieve precise cancer therapies, and one such promising strategy involves using nanoparticles (NPs) to enhance existing treatment methods [14,15]. This review highlights the advancements in the field of NPs for managing different malignant solid tumors over the last decade by referring to scientific literature and conclusions from multiple respectable journals.

## Review

NPs for the management of malignant solid tumors

NPs are solid entities ranging from 10 to 1,000 nm in size. They are also a rapidly emerging topic in nanotechnology as they offer a substantial potential resource for cancer care because they can encapsulate, trap, dissolve, or attach a range of medications. NP-based drug delivery systems have shown significant advantages in cancer management, including improved pharmacokinetics, targeted delivery, and reduced side effects and drug resistance [14,15]. Since the advent of nanotechnology, numerous nanotherapeutic drugs have been commercialized and are now widely available, with many more entering clinical trials since 2010. These nanotherapeutic agents have significantly advanced drug delivery systems and addressed antitumor multidrug resistance (MDR) issues, enabling combination therapies and targeting resistance mechanisms [16]. The initial exploration of nanotechnology in medicine began at the Federal Institute of Technology Zurich in the 1960s. This integration has proven effective in developing innovative diagnostic tools and enhanced treatment options [17].

**Figure 1:**
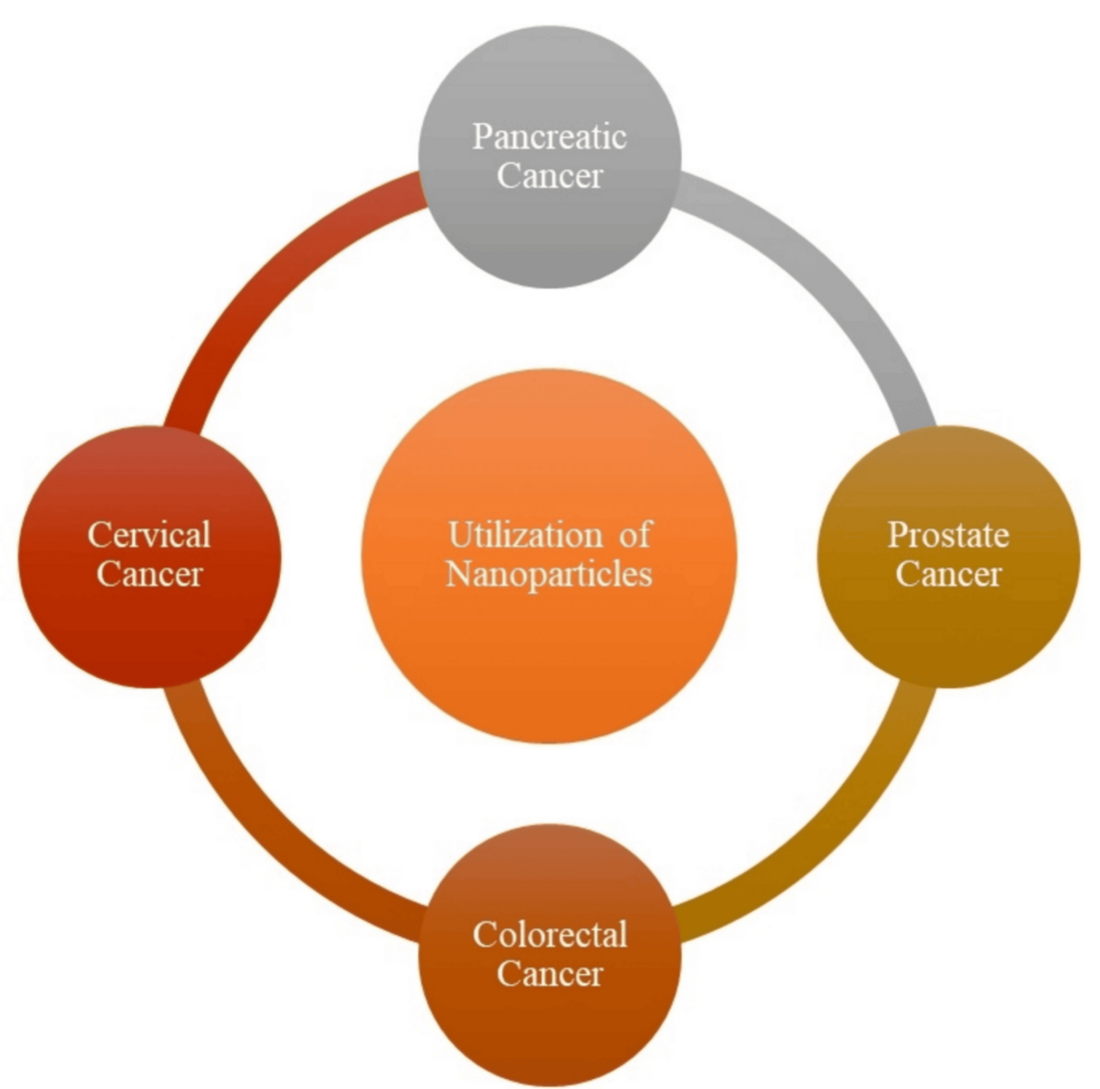
Utilization of NPs in cancer NPs: nanoparticles Image credits: The image was illustrated by the author Rohit R. Bhosale

Due to their remarkable characteristics, such as a high surface-to-volume ratio, diverse properties, submicrometer dimensions, and improved targeting capabilities, these materials have become increasingly significant across various multidisciplinary domains. Active targeting and passive targeting are the two main pharmacological targeting strategies involved. NPs are known for penetrating deep tissue, enhancing the permeability and retention (EPR) effect. Their surface properties significantly influence bioavailability and half-life by allowing effective passage through epithelial fenestrations [4]. For instance, NPs coated with polyethylene glycol (PEG), a hydrophilic polymer, can reduce opsonization and evade detection by the immune system. Furthermore, by adjusting the characteristics of the particle's polymer, it is possible to optimize the release rate of drugs or active compounds. Overall, the unique attributes of NPs play a crucial role in their effectiveness in cancer treatment and management [4,18-20]. A schematic of the utilization of NPs in various cancer types is shown in Figure 1.

The following are the highlights of the advancements in the field of NPs in managing different malignant solid tumors.

NPs for Pancreatic Cancer

Around 250,000 people die from pancreatic cancer (PC), a solid tumor with a high fatality rate each year worldwide. With a median diagnosis age of 71, it is most commonly diagnosed in people 40 years of age and older. Between one and ten PC instances are reported for every 100,000 people worldwide, with higher rates in rich countries [21]. PC ranks seventh globally in terms of the frequency of cancer-related deaths in both the male and female populations. However, the American Cancer Society reports that PC is currently the third most common cause of cancer-related mortality in the country [22,23]. PC is a rare and lethal cancer that mostly attacks the exocrine pancreas, with a five-year survival rate of less than 5%. These regions account for between 60% and 70% of these occurrences. Tumors in the body and tail regions occur less commonly (5%-10% and 10%-15% of PC cases, respectively) [24]. Microscopic PC tumors are surrounded by a dense stroma composed of ECM, internal fibroblasts, and inflammatory cells. Through paracrine and autocrine mechanisms, the intricate connections between tumor and stromal cells activate multiple signaling pathways, comprising matrix metalloproteinases, Wnt, transforming growth factor beta/small mother against decapentaplegic, Hedgehog, and hepatocyte growth factor/Met. Tumor invasion and growth are facilitated by the dynamic microenvironment created by these interactions [25].

Another characteristic that sets PC apart is its propensity to infect nearby tissues and spread to distant areas. PC management is based on the disease's stage at the time of diagnosis. Despite adjuvant therapy, the majority of patients relapse after surgery [26-28]. Due to late-stage diagnosis, early metastatic dissemination, lack of biomarkers for early screening, and the formation of systemic therapy resistance, PC has a dismal prognosis [28]. Treatments for a variety of tumor types, such as colorectal, prostate, cervical, and breast cancers, have significantly improved thanks to targeted drug delivery techniques that target particular genetic changes in cancer cells [29-31]. However, PC management remains difficult and yields poor-quality work. As a result, it is critical to share the most recent findings and significant conclusions regarding PC treatment with the scientific, medical, and research communities [32]. The neuronal microenvironment has a major role in the development of PC. In recent work, neuroscientists created ferritin NPs (Ft-NPs) intending to regulate the neuronal microenvironment to control the progression of PC. By binding to transferrin receptor 1, these Ft-NPs were engineered to actively and passively target PC malignancies by leveraging the tumors' EPR effects. The researchers loaded the brain activity activators CAB and ATO into Ft-NPs to produce nano-carbachol (CAB) NPs and nano-atropine (ATO) NPs, respectively. Pancreatic tumor growth was aided by nano-CAB NPs' efficient stimulation of the neuronal microenvironment. Conversely, nano-ATO NPs inhibited the neural niche, which hindered tumor neurogenesis and postponed PC development. For the focused distribution of neuroprotective drugs, ft-based NPs provide a stable and secure anticancer approach [33].

Additionally, researchers investigated the use of magnetic NPs (MNPs) and magnetic resonance imaging (MRI) to target the apoptotic inhibitor survivin (SUR) in a mouse model of pancreatic tumors. In the work, SUR antisense oligonucleotide (ASON) was conjugated with chitosan-coated MNPs to form SUR-MNPs. We evaluated the accumulation of NSON-MNPs (nonsense, targeted, and nontargeted oligonucleotide MNPs) in the liver, kidney, spleen, and tumor tissues. The data indicated that PC BxPC-3 cells exhibited a higher accumulation of targeted NPs compared to noncancerous cells. Additionally, tumors treated with nontargeted NPs, or NSON-MNPs, only slightly altered the signal. In a study involving mice, it was observed that tumors treated with targeted NPs exhibited a noteworthy decrease in T2 signal intensity during in vivo MRI [34].

In a different study, Prussian blue staining was used to verify that the tumor mass included a higher concentration of SUR-MNPs than the normal liver, pancreas, or kidney tissues. Given that ASON-functionalized MNPs are effectively localized to pancreatic tumors, these findings demonstrate the potential value of SUR-targeted NPs for pancreatic tumor detection [35]. Sensitive diagnostic instruments that can identify even tiny cancers at an early stage are desperately needed, as over 80% of PC patients pass away within the first six months of their diagnosis. To address this problem, magnetic biodegradable NPs were created by mixing iron oxide (maghemite, γ-Fe2O3) with recombinant human serum albumin. Since galectin-1 is highly expressed in PC but not in healthy or pancreatitis-related pancreatic tissue, it was chosen as the target receptor for this investigation. Tissue plasminogen activator-derived peptides covalently bonded galectin-1 to the surface of the NPs because of their high affinity for the target moiety. After these magnetic biodegradable NPs were given to the mice, single-photon emission CT and MRI demonstrated enhanced targeting and imaging performance [36].

Ultrashort echo-time (UTE) imaging has demonstrated potential in vivo for detecting tumor-targeted iron oxide NPs (IONPs) when paired with molecular MRI. One study evaluated how well MNPs targeted at receptors in cancer xenograft animals might be identified when employing positive contrast in UTE imaging. The tumor cells that expressed the epidermal growth factor receptor were the target of the ligands conjugated to the IONPs. The results showed that using both extended echo-time and ultrafast echo-time imaging techniques, PCs linked to the epidermal growth factor receptor showed high contrast [37]. Superparamagnetic iron oxide NPs (SPIONs) coated with polyethylenimine (PEI) have been studied for their potential use as transfection agents and their impact on the biology of tumor cells. It was unclear how PEI-coated SPIONs affected cancer cells in the pancreas. The study's findings demonstrated that these NPs significantly inhibited the migration and invasion of pancreatic tumor cells by significantly suppressing Src kinase activity and lowering the production of membrane type 1-matrix metalloproteinase and matrix metalloproteinase 2. In addition, after being treated with PEI-coated SPIONs, the density of the pancreatic tumor cell line Pan02 decreased. These generated NPs exhibited promising properties and could be applied to PC treatments as antimetastatic therapies [38].

Numerous investigations have been carried out to ascertain how diverse physiological situations impact the biological properties of NPs. A few characteristics of NPs that are heavily impacted by their physicochemical characteristics and biological environment are size, charge, and aggregation state. In biological contexts, proteins bind to the surface of NPs to form a protein coating that gives the particles a biological identity and regulates their physiological response. Trial participants included both healthy individuals and those with histologically proven PC. Notably, cancer patients' levels of therapeutically relevant proteins sharply dropped. The two patient groups received lipid NPs in two forms: one negatively charged (plain and PEGylated) and the other positively charged. The results showed that the zeta potential between the PC and healthy groups was considerably altered by simple positively charged lipid NPs [39].

A mathematical model of three phases was developed to represent drug release, degradation, diffusion, and relaxation. The model adequately described the release of PHT-427, an anticancer medication and kinase inhibitor, which was encased in poly(lactic-co-glycolic acid) NPs (PLGA NPs). The single emulsion-solvent evaporation approach was used in the study to encapsulate the protein kinase B/3-phosphoinositide-dependent protein kinase 1 inhibitor in NP form. The outcomes showed that the inhibitor had been properly sealed and delivered to the desired location [40]. Furthermore, the development of paclitaxel (PTX)-loaded mesoporous silica NPs (MSNPs) for intraperitoneal administration was a unique tactic. For an in vivo study, human PC cells (MIA PaCa-2) were injected into the peritoneal cavity of xenograft mice. The results demonstrated that MSNPs laden with PTX were absorbed into the systemic circulation more slowly and had a 3.2-fold longer residence duration in the peritoneal cavity. Compared to free PTX, PTX-loaded MSNPs showed a 6.5-fold increase in accumulation inside peritoneal tumors and a third of systemic exposure. Moreover, the cellular absorption of PTX-loaded MSNPs in tumor cells increased by a factor of 3.5. As a result, injecting MSNPs intraperitoneally reduced systemic exposure while increasing the amount of PTX gathered in peritoneal tumors [41].

In connection to the treatment of PC, the effects of cyclopamine, a potent inhibitor of the hedgehog signaling pathway with antifibrotic activity, on the efficacy and absorption of nanotherapeutics were investigated. To this end, cyclopamine NPs were synthesized, and the findings showed that they damaged extracellular fibronectins in tumors and enhanced tumor perfusion. Additionally, these NPs showed improved intratumoral dispersion and accumulation and relieved tumor vascular compression. Therefore, there is great anticipation that cyclopamine NPs would improve the effectiveness of treatment for PC patients [42]. Several scholarly articles and thorough evaluations have highlighted the higher fatality rate linked to PC. The growth of the stromal barrier, medication resistance, hypoxic conditions caused by hypoperfusion, and the presence of cancer stem cells are a few of these characteristics. To overcome these challenges, 5-fluorouracil (5-FU) and quercetin (QUER) were combined to form a nanocarrier system. Chitosan NPs were made by mixing and adding the two drugs separately. The results demonstrated a notable entrapment efficiency of the dual-drug-loaded carrier system, with a chitosan:QUER:5-FU ratio of 3:1:2. A strong link was found between the two drugs and the chitosan matrix. Additionally, in both 2D and 3D cultures, the dual-drug-loaded carrier system showed notable toxicity against PC cells [43].

Gemcitabine human serum albumin NPs (GEM-HSA-NPs) loaded with GEM were created and tested for their ability to inhibit the PC cell line BxPC-3 in vivo. According to the study's findings, GEM-HSA-NPs were more effective than free GEM because they showed greater drug loading and encapsulation [44]. Furthermore, the anticancer efficacy of doxorubicin-loaded gold NPs (DOX-GNPs) was evaluated using a green chemistry approach against human PC cell lines. Following its description and use in an in vitro anticancer experiment, the synthesized DOX-GNPs in the PC cell lines did not exhibit any appreciable change in percentage cell viability compared to DOX [45]. Consequently, NPs are emerging as a promising approach to combat PC [46].

NPs for Prostate Cancer

With 258,000 fatalities from prostate cancer in 2008, it is the second most frequent malignancy worldwide and the leading cause of death [30]. It is a condition that is diagnosed regularly worldwide in men. While over 417,000 cases of prostate cancer were reported in Europe in 2014 and 2015, the United States alone was predicted to have 230,000 and 280,000 cases in 2014 and 2015, respectively [47,48]. Over the past 20 years, there has been a dramatic rise in the survival rate for different stages of prostate cancer, going from 69% to over 99%, mostly due to earlier diagnosis, more public awareness, and pharmacological advancements. However, African American males have a death rate from prostate cancer that is more than twice as high as that of Caucasian men [49,50]. Family history, age, and race are important risk factors; however, interactions between endogenous and environmental factors also have a role in the development of prostate cancer [51]. The type of cancer will determine the available treatment choices for prostate cancer. Radiation therapy or surgery can effectively treat localized prostate cancer, but metastatic prostate cancer is incurable. When castration resistance develops, chemotherapy becomes the preferred course of treatment, prolonging the patients' life by several months [52].

The use of nanotechnology in prostate cancer treatment has several advantages. For prostate cancer active targeting and imaging, thermosensitive magnetic NPs (PMNPs) coated with poly(N-isopropylacrylamide-acrylamide-allylamine) were developed in one study. These NPs were used to conjugate further R11 peptides (R11-PMNPs), which are unique to prostate cancer. After being incubated for 24 hours, the NPs showed signs of being superparamagnetic. At dosages of up to 500 μg/mL, they were also compatible with normal prostate epithelial cells and human dermal fibroblasts. Moreover, PC3 and LNCaP prostate cancer cells absorbed R11-PMNPs more easily than PMNPs. In contrast to PMNPs lacking R11 conjugation, R11-PMNPs accumulated more in tumors than in other essential organs, per in vivo biodistribution studies [53].

Another strategy was encapsulating curcumin (CUR) in a nanoparticulate structure that might scavenge radicals, shielding it from oxidative degradation. Nitroxide radicals, which function as reactive oxygen species (ROS) scavengers, were coupled with self-assembling amphiphilic block copolymers to form pH-sensitive redox NPs loaded with CUR (RNPN). Higher bioavailability and considerable ROS scavenging at the tumor locations explained the new CUR-loaded RNPN system's lowered in vivo tumor development [54]. Researchers looked at the possibility of using PLGA-CUR NPs as a therapy for prostate cancer. The effective uptake of these NPs into prostate cancer cells and the subsequent release of active CUR in the cytosolic region led to an improved therapeutic effect. In studies for cell proliferation, the generated NPs dramatically decreased prostate cancer cell proliferation and colony formation capability when compared with natural CUR [55].

Docetaxel (DTX) is a highly successful therapy for metastatic castration-resistant prostate cancer, which may enhance the quality of life and increase survival. However, long-term usage is limited by cumulative toxicity and the development of drug resistance. DTX was evaluated via superficial conjugation to carboxymethylcellulose NPs to overcome these limitations. Remarkably, mice's PC3 tumor xenografts vanished entirely after ingesting one dose of our synthetic NPs [56]. It has been established that multiple myeloma and prostate cancer cell lines are not susceptible to the antitumor effects of *Walterinnesia aegyptia* (WEV) snake venom. Using mice as models, the therapeutic efficacy of the extracted venom after treatment with silica NPs (WEV+NP) was assessed. Prostate tumor volumes were significantly reduced, ROS were elevated, and chemokine levels dynamically decreased when prostate tumor volumes were treated with WEV+NP. These results indicate the potential for delivering snake venom to prostate cancer cells over a prolonged period using the developed nanoparticulate technology [57].

Gold NPs (AuNPs) offer a versatile nanomaterial platform for use in biological research. In the context of prostate cancer, AuNPs were developed and functionalized particularly to target the prostate-specific membrane antigen (PSMA), which is generated by prostate cancer cells. Streptavidin-coated AuNPs conjugated with a PSMA inhibitor exhibited robust and specific binding to PSMA-expressing LNCaP cells in contrast to nontargeted AuNPs. The potential of this technology for customized delivery of prostate cancer treatment was demonstrated [58]. To create flutamide (FLT)-loaded casein NPs (CASNPs), FLT was first encapsulated in CASNPs. The objectives were to reduce hepatotoxicity, improve anticancer activity, and manage drug release. When CASNPs were injected intravenously for 28 days, they showed increased antitumor activity in prostate cancer-bearing rats and sustained drug release for up to four days. Prostate-specific antigen serum levels significantly dropped due to these NPs, and the relative weights of prostate cancer decreased as well. In addition, compared to the medication solution, they demonstrated increased antiproliferative, antiangiogenic, and apoptotic effects [59]. NPs have demonstrated great promise for treating prostate cancer by offering better drug administration, enhanced therapeutic effectiveness, and fewer adverse effects.

NPs for Colorectal Cancer

Cancer has become one of the biggest threats to human health due to an increase in incidence worldwide; in 2018, there were 9.6 million deaths, and 1.8 million reported new cases [60,61]. As the third most prevalent type of cancer globally, colon cancer is a major cause of death, especially in the United States [62,63]. The accumulation of genetic and epigenetic alterations is thought to be responsible for the genesis of colon cancer and its subsequent progression. However, the early signs of colon cancer are frequently mild and only show up in the middle and advanced stages of the disease. They can include weight loss, anemia, and blood in the stool [64]. Lifestyle factors that contribute significantly to tumor formation include smoking, eating poorly, and not exercising. Moreover, cytokines, growth hormones, and certain chemicals are among the elements that facilitate the advancement of colon cancer [65]. Globally, colorectal cancer is now a lethal illness due to a lack of efficient drug delivery strategies and early detection techniques. In this regard, nanotechnology has become a viable strategy, especially for drug delivery that targets diseased locations more successfully. Using NPs in colon cancer imaging and therapy has drawn much interest [66-68].

In one study, 5-FU was encapsulated in biodegradable polycaprolactone NPs and found to be more effective than other NPs, as well as the drug solution, for stopping the growth of colorectal cancer cells [69]. Another study demonstrated the anticolon cancer potential of chitosan-based NPs loaded with 5-FU and epigallocatechin-3-gallate, mediated via wheat germ agglutinin. These NPs demonstrated extended circulation times and improved medication localization at the tumor location [70]. Since MNPs have specific physicochemical characteristics that enable customized interactions with cells and biocompatibility, they have also demonstrated potential as nanocarriers in biomedical applications. For example, following oral medication, the tumor microenvironment-mediated extrinsic signals enabled dextran-coated superparamagnetic iron oxide solid lipid NPs loaded with DOX and adorned with folate residues to accumulate DOX at colon tumor sites preferentially [71]. A different formulation used solid glyceryl trimyristate-based magnetic solid lipid NPs with iron oxide centers. When human HT29 colon cancer cells were exposed to alternating electromagnetic fields, their vitality decreased [72].

By interfering with cellular connections and the cell cycle, biocompatible polymer/carbohydrate-coated MNPs could inhibit colon cancer cells by 20% [73]. Compared to radio-labeled resveratrol alone, AuNPs incorporating radio-labeled resveratrol demonstrated increased targeted efficacy on colon cancer cells in rats [74]. Because of the photothermal characteristics of AuNPs, hyaluronic acid and SN38-loaded AuNPs showed site-specific targeting effects that effectively inhibited cancer cell proliferation [75]. In colon cancer cell cultures, NPs made of chitosan and PEGylated chitosan, encapsulated with anti-catenin small interfering RNA, and produced using the ionic gelation process effectively reduced the levels of catenin protein [76]. Furthermore, green-synthesized abutilon indicum leaf extract-encapsulated AuNPs with polyphenol stabilization demonstrated strong lethal effects on cancer cells [77]. Using N,O-carboxymethyl chitosan, combinatorial NPs encapsulating the well-known anticancer phytochemicals 5-FU and CUR were created. This led to higher drug levels in the bloodstream and improved treatment efficacy for colon cancer [78].

In recent work, the anticancer medication DOX was synthesized as MSNPs functionalized with an aptamer that targets the epithelial cell adhesion molecule. Due to this adjustment, DOX may be given to colon cancer cells only, improving its therapeutic index and lowering its negative effects [79]. The study of simultaneous cancer detection and therapy, or theranostics, is a rapidly emerging field. One significant achievement is the creation of multifunctional branching glycopolymer-PTX-dodecane tetraacetic acid-gadolinium ion NPs, which have great potential for cancer theranostics. These NPs showed a significant decrease in tumor development, enhanced MRI contrast intensity, and enhanced biocompatibility [80]. As a result, NPs are becoming an exciting avenue for fighting colorectal cancer. Their unique properties offer new possibilities for treatment in this area.

NPs for Cervical Cancer

Cervical cancer is the second most common cause of death for women worldwide [29]. The lack of established screening and early detection programs is the reason behind the high incidence of cervical and breast cancer in developing nations, as well as in America and Africa. Cervical cancer was once thought to be mostly caused by the human papillomavirus (HPV). However, HPV, the primary etiological factor, was established as real and unquestionably present after 2000. The HPV virus produces proteins that alter the retinoblastoma and P53 genes, among other important genes that control cell growth. These proteins also impede programmed cell death or apoptosis. Cervical cancer risk factors include HIV infection, having numerous sexual partners, and having multiple sexual partners [81-83].

Smoking is also harmful because it impairs immunity, which promotes the development of cancer. Squamous cell carcinoma, adenocarcinoma, villoglandular adenocarcinoma, melanoma, and adenosquamous carcinoma are among the several types of cervical carcinomas. Cervical cancer usually begins with abnormal cells growing on the surface of the cervical cavity and advances gradually. Squamous cells are the source of many cervical malignancies, which can result in dysplasia, a precancerous disease [84]. Cervical cancer and/or precancerous lesions are most common in women who have ongoing HPV infections [85-87]. Therefore, the research and scientific communities must know the most recent advancements and significant findings in managing cervical cancer. Because they can act as a reservoir for medications and make it easier for them to be encapsulated, trapped, dissolved, or attached to an NP matrix, NPs have the potential to completely transform the way cancer is treated [88,89].

With a cellular absorption of 81% higher than that of plain gelatin NPs (51% uptake), cisplatin showed better drug delivery capabilities when encased in folic acid-conjugated gelatin NPs for cancer treatment [90]. Using the nanoprecipitation process, NPs containing the bioflavonoid naringenin showed improved cytotoxic activity against human cervical cancer cells. This treatment was superior to free naringenin based on increased intracellular ROS, lipid peroxidation, and decreased glutathione levels [91]. The oligonucleotide intercalator phenanthridinium-functionalized MSNPs, especially in HeLa cells, exhibited considerable growth inhibition by binding to cytoplasmic oligonucleotides thanks to the phenanthridium groups on their surface. These NPs demonstrated promising cellular trafficking characteristics, strong biocompatibility, and a range of biomedical uses [92].

*Podophyllum hexandrum* green leaf extract generated silver NPs that preferentially broke down DNA and triggered caspase-mediated cell death in human cervical cancer cells [93]. Podophyllum hexandrum L-derived crystalline AuNPs have demonstrated a strong anticancer impact by generating DNA damage, oxidative stress, cell cycle arrest, and caspase cascade activation [94]. In the end, this caused cancer cells to become dysfunctional and die. Nanostructured lipid particles laden with bleomycin sulfate enhanced its oral bioavailability while simultaneously increasing intestinal lymphatic absorption and inhibiting first-pass metabolism. Among the results of this were increased toxicity and apoptosis against cervical cancer cells [95]. Anti-P-glycoprotein antibody was coupled to PLGA NPs loaded with CUR to increase their solubility and stability. These NPs demonstrated potential as anticancer medications or as modulators of MDR, helping cancer patients [96].

Many studies have looked into the usage of DOX in the treatment of cervical cancer. One work investigated the production of long-circulating self-assembled NPs encased in DOX. An amphiphilic block copolymer of poly(ethylene glycol) and polynorbonene-cholesterol was used to create these NPs. Compared to free DOX, these NPs greatly reduced the growth of tumors. According to the findings, these NPs may be useful for delivering hydrophobic anticancer medications to tumors [97]. The use of MSNPs loaded with DOX hydrochloride and coated with a pH-responsive charge-reversal polymer was examined in another investigation. These NPs effectively carried and released DOX hydrochloride into the nucleus of HeLa cells, as demonstrated by confocal laser scanning imaging [98]. Furthermore, cyclodextrin was added to a pH-sensitive poly(2-(dimethylamino)ethyl methacrylate) star polymer to produce DOX-loaded NPs. These NPs demonstrated enhanced cytotoxicity and cellular absorption, and they effectively stopped tumor growth without having any discernible negative effects [99]. Researchers used an aqueous extract from brown seaweed to manufacture magnetic IONPs. These NPs induce apoptosis in cervical cancer cells, which has lethal consequences [100]. Targeted NPs based on transferrin-conjugated amphiphilic copolymers loaded with PTX showed enhanced efficacy and enabled tumor-specific treatment because of the presence of transferring agents [101].

Using emulsion solvent evaporation and simple dialysis with poly(lactide-co-glycolide) and Pluronic F68, biocompatible amphiphilic pentablock copolymeric NPs loaded with DTX were created. When these NPs were administered to cervical cancer cells, they demonstrated significant cytotoxicity [102]. When methotrexate and 5-FU were used together in NPs, a higher antitumor efficacy was observed compared to when either drug was administered individually or when the NP formulations were administered separately [103]. Additionally, Table 1 briefly represents the diverse NPs synthesized very recently for managing different types of malignant solid tumors reported in this review. Consequently, nanotechnology has the significant and exciting potential to design, develop, and innovate new and advanced pharmaceutical delivery methods for cancer management since it offers a variety of opportunities. Thus, NPs are emerging as a fascinating strategy for addressing cervical cancer.

**Table 1:** Different types of NPs synthesized recently for managing different types of malignant solid tumors reported NPs: nanoparticles; SeNPs: selenium nanoparticles; PC: pancreatic cancer; VISTA: V-domain Ig suppressor of T cell activation; ZnONPs/CS: zinc oxide nanoparticles/chitosan; PLGA: poly(lactide-co-glycolide); AgCa-NPs: agar-encapsulated carnosine nanoparticles; mPEG-PLA-NPs: polymeric polyethylene glycol-polylactic acid nanoparticles; MOLP-AgNPs: *Moringa oleifera* leaf powder-silver nanoparticles; AgNPs: silver nanoparticles

Type of NPs	Drug	Cancer type	Study
SeNPs	Novel heteroxylan PVP3-1, extracted from cluster of *Prunella vulgaris* L.	PC	Zhang et al. [104]
Fe_3_O_4_@TiO_2_ NPs	VISTA monoclonal antibodies	PC	Hong et al. [105]
Enzyme-responsive aminoethyl anisamide-modified NPs	Squalenoyl-chidamide prodrug	PC	Chen et al. [106]
Gemcitabine-phospholipid complex-loaded lipid NPs	Gemcitabine	PC	Dora et al. [107]
β-cyclodextrin-magnetic graphene oxide NPs	Curcumin	Prostate cancer	Einafshar et al. [108]
ZnONPs/CS	Caffeic acid phenethyl ester	Prostate cancer	Ince et al. [109]
Deer antler extract NPs	Deer antler water extract	Prostate cancer	Nazarbek et al. [110]
PLGA NPs	Brusatol and docetaxel	Prostate cancer	Adekiya et al. [111]
AgCa-NPs	Carnosine	Colorectal cancer	Hsieh et al. [112]
mPEG-PLA-NPs	Epirubicin	Colorectal cancer	Khan et al. [113]
MOLP-AgNPs	MOLP	Colorectal cancer	Susanto et al. [114]
AgNPs	*Catharanthus roseus* leaf extract	Cervical cancer	Hussein et al. [115]
	Arabic gum	Cervical cancer	Li and Gao [116]
	Caffeic acid	Cervical cancer	Pushpanathan et al. [117]

Potential and regulatory challenges of NP-based therapies

The production of NPs involves several complex challenges. First, many chemical methods used in their creation can have adverse environmental effects, demanding stringent waste management practices to address these risks. Moreover, NPs can be hazardous, particularly when utilized in medical contexts, necessitating thorough safety and efficacy evaluations to ensure they are safe for use. Consistency in particle size is another issue, especially during large-scale manufacturing. While some eco-friendly technologies might work on a smaller scale, they often encounter difficulties when scaled up. Understanding the precise synthesis pathways for NPs remains problematic. The lack of a clear grasp of these processes makes it hard to reliably standardize or replicate production methods. Combining different metals with plant materials adds another layer of complexity. Each metal requires specific conditions and tailored technological approaches for effective synthesis. Furthermore, the absence of established regulatory standards for NP-based drugs leads to uncertainty and complications in meeting required safety and efficacy benchmarks [118].

Translational barriers and challenges in the clinical translation of NP-based therapies for cancer treatment

From a researcher's perspective, it's disheartening to see that much of the foundational research has not successfully transitioned into clinical practice. A quarter-century after the introduction of Doxil, the first nano-chemotherapeutic agent, the number of approved nanomedicines for cancer treatment remains surprisingly low. Despite numerous research efforts to leverage NPs for cancer therapies, only a handful of formulations have advanced beyond the clinical trial stage. A deep look into NP-based products available and those in trials reveals a scarcity of nanoformulations that have received market approval for cancer treatment. Moreover, the U.S. Food and Drug Administration (FDA) has approved very few nano-based cancer drugs, while most nanoformulations are still undergoing clinical trials [119,120].

Despite significant advancements, numerous challenges hinder the progression of nanodrugs from clinical settings to the marketplace. A primary obstacle is the insufficient understanding of how NPs interact with biomolecules, complicating their development. Moreover, consistently producing NPs that effectively target cancer cells can pose difficulties for pharmaceutical companies. There are several instances, such as with DepoCyt, where companies have halted drug development, often citing technical challenges as the reason. Researchers and pharmaceutical firms face various hurdles before a drug can be deemed viable for cancer treatment. Consequently, many nanoformulations have been withdrawn from the market even after receiving FDA approval. Examples include Feridex IV (Endorem, Ferumoxides), Clariscan (PEG-Fero, Feruglose NC100150), GastroMARK, Resovist (Cliavist), Lumirem (GastroMARK), and Sinerem (Combidex) [121,122].

Several factors contribute to the challenges of translating research findings into marketable products. These include difficulty predicting patient susceptibility to allergic reactions, accurately quantifying endotoxins, ensuring effective cellular uptake of the drug, achieving sustained drug release, and overcoming biological barriers. Additionally, enhancing the drug's ability to specifically target tumor cells and managing the immune system's response to new therapies are significant hurdles that researchers face [123-134]. Challenges in the clinical translation of NPs in cancer therapeutics include the lengthy drug development process and the significant time required for preclinical and clinical studies involving larger animals and human participants. Obtaining regulatory approval for market release can also be complicated. Additionally, there are issues related to effectively loading drugs into NPs, formulation instability, and concerns about biocompatibility and toxicity. Other challenges include insufficient time for the drug to remain active in the body, difficulties in ensuring that the formulation accumulates at the tumor site, and issues with drug loading, internalization, and release. Moreover, incomplete biodegradation and elimination, problems with cellular uptake, and the struggle to translate successful in vitro results to in vivo applications further complicate the process [135-145].

Future perspectives of NPs for cancer management

Nanotechnology is revolutionizing cancer treatment by advancing the precision delivery of therapeutic molecules for diagnosing and treating the disease. NPs are increasingly utilized in various cancer therapies due to their unique properties. These offer notable advantages over conventional medications, including enhanced pharmacokinetics, improved biocompatibility, better tumor targeting, and greater stability. NPs also provide a flexible platform for combining different therapies, which can significantly boost treatment outcomes. Recent research has highlighted the effectiveness of different NP types, such as polymeric, metallic, and hybrid NPs, in enhancing drug delivery. It is essential for scientists to thoroughly understand both the features of these nanoplatforms and the specific attributes of the drugs they transport. Furthermore, integrating immunomodulatory agents into NPs might improve vaccine performance in immunotherapy. Despite the exciting potential, the actual use of NP-based drugs is still limited, with only a few approved for clinical use, some undergoing trials, and many more in the exploratory stages. To advance the field, additional research is needed to tackle toxicity concerns and gain a deeper insight into how cellular and physiological factors affect NPs behavior within the human body [4,145].

## Conclusions

The administration of anticancer medications is one of the many biological applications for which nanocarriers is a ground-breaking scientific discovery. When it comes to therapeutic efficacy, prolonged circulation length, repeat administration, and targeted and regulated release of the medication in response to specific stimuli, nanocarriers have shown to be a significant advance over conventional cancer treatment. Because of this, drugs can be precisely formulated to release from nanocarriers, such as NPs, at the right place inside the body. There is great promise for improving cancer therapy through development and utilization of NPs in diagnostic and treatment approaches, thereby holding huge potential to be extensively used for efficient cancer therapy with an additional development and invention.
